# Intrapleural Fibrinolytic Therapy Improves Results With Talc Slurry Pleurodesis

**DOI:** 10.7759/cureus.10122

**Published:** 2020-08-29

**Authors:** Alyssa Bellini, Francisco Tarrazzi, Catherine Tami, Sanja H Patino, Mark Block

**Affiliations:** 1 Department of Surgery, University of California Davis, Sacramento, USA; 2 Division of Thoracic Surgery, Memorial Healthcare, Hollywood, USA; 3 Internal Medicine, Florida Atlantic University Charles E. Schmidt College of Medicine, Boca Raton, USA

**Keywords:** talc slurry pleurodesis, talc pleurodesis, tpa, tissue plasminogen activator, pleural effusion, loculated pleural effusion

## Abstract

Objective

Talc slurry pleurodesis (TSP) can lead to permanent small loculations. Intrapleural tissue plasminogen activator (tPA) breaks down loculations, and therefore may improve results but may also inhibit pleurodesis. tPA was given with and after talc slurry to promote more uniform talc distribution and eliminate loculations.

Methods

Charts were reviewed for patients treated with TSP with or without tPA. Chest x-rays after TSP were compared to chest x-rays before and graded as “worse”, “same”, or “better”. Incidence of need for repeat TSP was recorded.

Results

There were 52 patients, eight with bilateral effusions, for a study cohort of 60 effusions. One-third of the effusions were malignant. No patients experienced significant bleeding. Results were better than baseline for 14 (26%) patients given tPA, but not for patients that never received tPA. The addition of tPA 4-6 mg with talc slurry resulted in no patients requiring repeat TSP. When tPA was given after talc slurry, a delay of three days was associated with the lowest incidence of repeat TSP (3/14, 21%).

Conclusions

There were no significant complications from tPA use to supplement TSP, and tPA may improve results without interfering with pleurodesis. A prospective trial is warranted.

## Introduction

Recurrent pleural effusion is a common clinical problem, with an estimated incidence of 1.5 million per year in the United States [1]. If optimal management of the underlying medical condition does not resolve the effusion, then options for palliation are intermittent thoracentesis, indwelling pleural catheter, or pleurodesis. Chemical pleurodesis causes an inflammatory response that produces fibrin adhesion and fibrosis, obliterating the pleural space [2]. Of the many agents available for chemical pleurodesis, a Cochrane Review concluded that talc is the agent of choice [3]. Bedside pleurodesis (talc slurry) is easier to perform and less costly than operative pleurodesis (talc poudrage), and may yield similar outcomes [4-6]. A critical concern is that uneven distribution of talc may lead to uneven pleurodesis and a multiloculated effusion [7-9].

Tissue plasminogen activator (tPA) is a fibrinolytic that has been shown to be effective for drainage of loculated pleural effusions [10-12]. Based on our favorable experience with intrapleural tPA for management of complex pleural effusions [13], we started using it to eliminate loculated collections that developed after pleurodesis. To the best of our knowledge, this had not been attempted before. Our initial experience was promising and safe, and therefore, our service started the routine use of tPA to supplement talc slurry pleurodesis (TSP), giving it either with and/or after talc slurry. We hypothesized that administration with talc slurry would improve distribution of talc throughout the pleural space, and that it would break down loculated collections if given after talc slurry. The potential downside of giving it after talc slurry was that it might interfere with effective pleurodesis, and thus, dosing and timing were variable. This report is a retrospective analysis to determine if objective findings support the continued use of tPA to supplement TSP, and if a more rigorous prospective investigation is warranted.

## Materials and methods

Institutional Review Board approval was obtained for this retrospective review. Charts were reviewed for patients treated with TSP for recurring pleural effusions between March 2014 and December 2016. Demographics and clinical data were collected, and imaging results recorded.

Technique of talc slurry pleurodesis

All patients had pigtail chest tubes (sizes 8-14 Fr) inserted under image guidance for recurring pleural effusion. TSP was performed by injection of talc slurry (5 mg sterile asbestos-free talc mixed with 20 mg lidocaine 1% in 60 ml normal saline) through the chest tube and then clamping the tube for 6 hours. Beginning in April 2014, tPA (2-6 mg) was also given through the chest tube either with the talc slurry and/or at an interval after. Patients were followed daily for chest tube output and with imaging (chest x-ray and/or chest CT). Management decisions regarding when to remove the chest tube, whether to administer additional tPA, or whether to repeat TSP were made by the clinician based on daily evaluations and did not follow a clearly defined protocol.

Assessment of results

Results of TSP were determined based on imaging and the need for repeat pleurodesis. Qualitative outcome assessments (“worse”, “same”, “better”) were made by comparing the end-result imaging (chest x-rays after TSP at the time of chest tube removal) to chest x-rays showing the best lung expansion before starting pleurodesis (after chest tube placement but before TSP). Duration of chest tube drainage was also recorded.

Statistics

Statistical analyses were not performed because this is a retrospective review with a high degree of variability in treatment decisions for each patient, and therefore sample sizes for groups with similar treatment are relatively small. Furthermore, the intent of the review is to determine if there is sufficient evidence to warrant a statistically valid prospective evaluation, rather than to definitively evaluate efficacy.

## Results

Fifty-seven patients had TSP for recurring pleural effusion, of which five either expired or were discharged to hospice within four days, and thus, were excluded from analysis. Eight of the remaining 52 patients had bilateral TSP for bilateral pleural effusions, yielding a study cohort of 60 interventions. Of these, seven effusions were treated with talc slurry without tPA (“TSP without tPA”), and 53 effusions were treated with talc slurry and tPA (“TSP with tPA”; Table 1). Heart failure and cancer each accounted for slightly more than a third of the effusions. Gender distribution was approximately equal and most effusions (65%) were right-sided. No patients experienced bleeding in response to tPA sufficient to cause hemodynamic instability.

**Table 1:** Patient Characteristics TSP: talc slurry pleurodesis, tPA: tissue plasminogen activator, CHF: congestive heart failure, ESRD: end-stage renal disease

Characteristic	All	TSP without tPA	TSP with tPA
Total cases, n	60	7	53
Mean age, years; (range)	73	77 (58-90)	72 (37-99)
Sex, n			
Male	27 (45%)	5 (71%)	22 (42%)
Female	33 (55%)	2 (29%)	31 (58%)
Etiology, n			
CHF	22	4	18
Malignant	21	2	19
Cirrhosis	8	1	7
ESRD	8	0	8
Sickle cell	1	0	1
Laterality, n			
Right	38	5	33
Left	6	0	6
Bilateral	8	1	7

**Figure 1:**
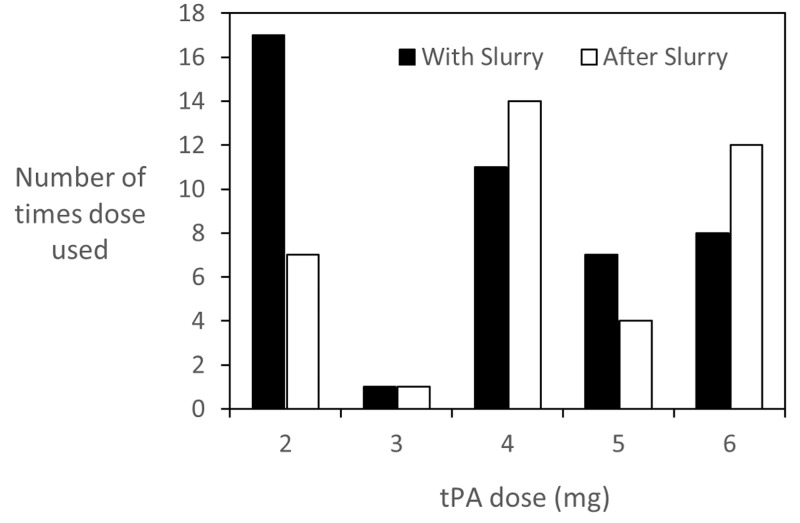
Frequency of use of different doses of tissue plasminogen activator (tPA). The dose of tPA given both with the slurry and after the slurry varied from 2-6 mg. The number of times each dose was used is shown, with empty bars indicating doses given with talc slurry, and filled bars doses given after talc slurry.

Results of TSP with and without tPA were assessed by evaluating radiographic results, incidence of pleurodesis failure (as measured by need for repeat talc slurry), and duration of chest tube drainage. The proportion of patients with a satisfactory (“same or better”) radiographic result was similar whether or not tPA was used (72% “Any tPA” vs. 71% “Without tPA”), but tPA use was associated with improvement (“better”) in 26% of cases (Figure 1), compared to no improvement in cases in which tPA was not used (Table 2). At the same time, tPA use was not associated with a higher incidence of pleurodesis failure, as measured by the need for repeat TSP (57% of cases not receiving tPA compared to 43% of cases receiving tPA). Among the effusions treated with TSP and tPA, the lowest rate of failure was seen in the group that received tPA only after talc slurry (33%), and the highest rate was in the group that received tPA both with and after talc slurry (48%). Although tPA use was associated with a one day longer median duration of chest tube drainage compared to no tPA (nine days vs. eight days), when tPA was given only with talc slurry median duration was shorter (five days). Maximum chest tube duration was much longer when tPA was given (35 days vs. 10 days), reflecting ongoing management with tPA dosing and repeat talc slurry administration in that one patient. The frequency of use of various doses of tPA, from 2-6 mg, is shown in Figure 1.

**Table 2:** Results of TSP with or without tPA TSP: talc slurry pleurodesis, tPA: tissue plasminogen activator

		Chest x-ray Result				Number needing repeat TSP, N (%)	Chest tube duration (days), Median (range)
Group	N	Worse, (N)	Same , (N)	Better, N(%)	Same or better, N (%)		
Without tPA	7	2	5	0 (0)	5 (71)	4 (57)	8 (3-10)
Any tPA	53	15	24	14 (26)	38 (72)	23 (43)	9 (2-35)
Only with Slurry	15	4	6	5 (33)	11 (73)	6 (40)	5 (2-18)
Only after Slurry	9	2	5	2 (22)	7 (78)	3 (33)	9 (5-31)
With & After Slurry	29	9	13	7 (24)	20 (69)	4 (48)	12 (5-35)

To evaluate the hypothesis that giving tPA with talc slurry might improve talc distribution and mesothelial contact, we looked at results in the 15 cases where tPA was given only with talc slurry (Table 3). The lowest dose (2 mg) was associated with a high proportion of satisfactory radiographic results (78%), but also a high incidence of pleurodesis failure (67%). In contrast, the higher doses of 4-6 mg were associated with a high proportion of satisfactory results (67%) and no pleurodesis failures.

**Table 3:** Effect of tPA dose when given with talc slurry TSP: talc slurry pleurodesis, tPA: tissue plasminogen activator

tPA Dose (mg)	N	Chest x-ray Result				Number needing repeat TSP, N (%)
		Worse, N	Same, N	Better, N	Same or better, N (%)	
2	9	2	3	4	7 (78)	6 (67)
4	3	1	2	0	2 (57)	0
5	1	1	0	0	0	0
6	2	0	1	1	2 (100)	0

**Figure 2:**
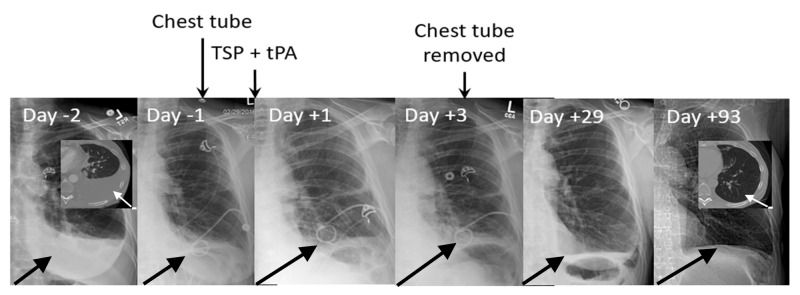
TSP + tPA for recurrent malignant pleural effusion. A 60-year-old woman 17 years post right upper lobectomy and 12 years post radio-frequency ablation for separate primary lung cancers, presented with a recurrent left malignant pleural effusion (day -2). A pigtail chest tube was inserted on day -1 and TSP performed with tPA, 4 mg, on day 0. The chest tube was removed on day +3. Follow-up imaging showed no recurrence (days +29 and +93). TSP: talc slurry pleurodesis, tPA: tissue plasminogen activator

**Figure 3:**
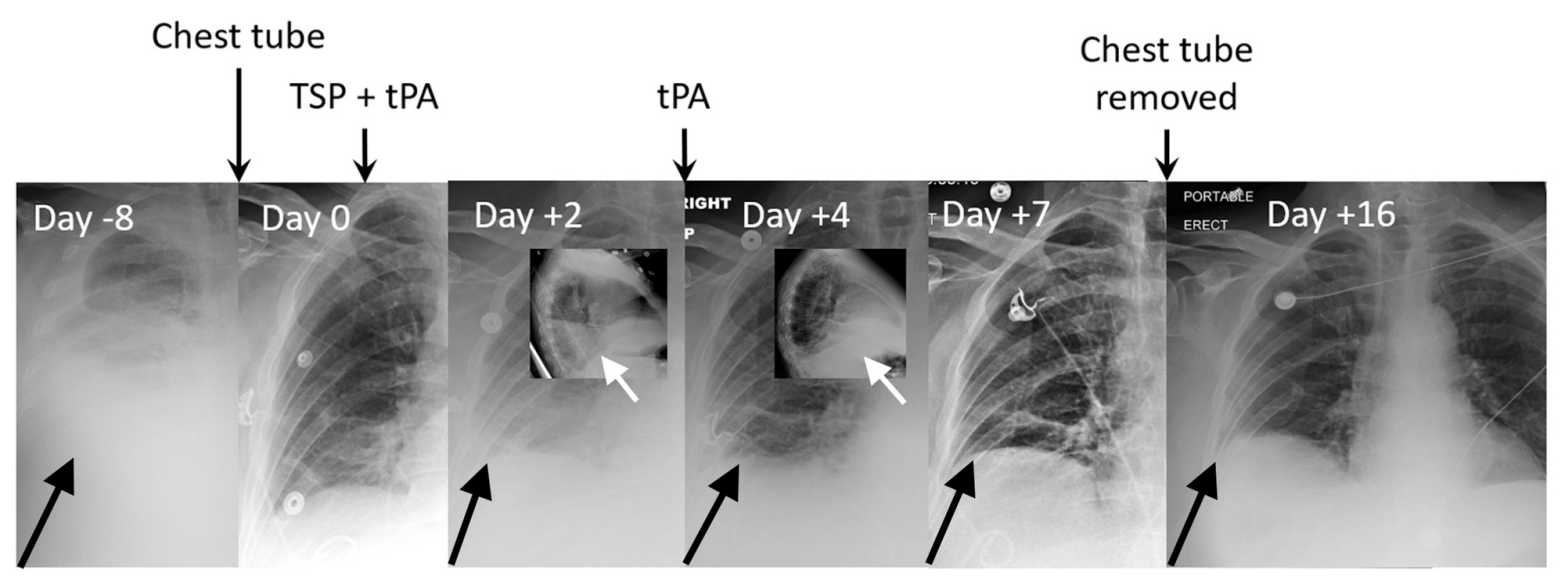
tPA after TSP for drainage of developing loculation. A 67-year-old man presented with hepatic hydrothorax (day -8). A pigtail chest tube was placed with output >2,000 cc/day. TSP with tPA, 6 mg, was given on day 0. Lateral chest x-ray on day +2 demonstrated accumulating loculation so tPA, 6 mg, was given on day +3. Lateral chest x-ray on day +4 demonstrated resolution of the effusion. Chest tube removed on day +8 with follow-up chest x-ray on day +16 showing resolution. TSP: talc slurry pleurodesis, tPA: tissue plasminogen activator

The concern with giving tPA after talc slurry is that the fibrinolytic effect might interfere with effective pleurodesis, and therefore, we evaluated the effect of timing between talc slurry and tPA on the incidence of TSP failure (Table 4). Without tPA, the failure rate was 57% (Table 2), and when tPA was given only with talc slurry it was 40%. When given after talc slurry, the lowest rate of pleurodesis failure was seen with an interval of three days (21%) (Figure 2 and Figure 3), while intervals of five days or more were associated with a very high 82% failure rate.

**Table 4:** Effect of interval between talc slurry and tPA on incidence of repeat TSP TSP: talc slurry pleurodesis, tPA: tissue plasminogen activator

Interval to First Dose of tPA (days)	N	Incidence of Repeat TSP N (%)
0	15	6 (40)
1	8	3 (37)
2	3	1 (33)
3	14	3 (21)
4	2	1 (50)
≥5	11	9 (82)

## Discussion

Despite the growing popularity of indwelling pleural catheters for management of recurrent pleural effusion, pleurodesis remains an important option. Patients may not be able or willing to care for an indwelling catheter, or the effusion may be the result of a chronic condition, such as heart, liver or renal failure, in which case spontaneous pleurodesis with an indwelling catheter is unlikely. In these circumstances, chemical pleurodesis may be the best option. Talc has been the preferred agent, and although intraoperative administration as talc poudrage is the traditional technique, good clinical evidence indicates that bedside talc slurry is equally effective [4-6]. Unfortunately, as of this writing, sterile talc for pleurodesis is no longer available. Nevertheless, the underlying physiology of pleurodesis should be the same regardless of agent, and therefore, strategies to optimize results with TSP are relevant for bedside chemical pleurodesis with other agents.

An important deterrent to the use of pleurodesis is that results are sometimes suboptimal. Patients often develop residual loculated effusions that can become permanent if not aggressively drained, and occasionally, pleurodesis is incomplete, requiring re-administration of the pleurodesis agent. Several strategies have been used to address these problems. For example, the argument in favor of talc poudrage is that intraoperative administration optimizes delivery of talc throughout the pleural space, minimizing the likelihood of developing loculations [7-9]. However, clinical studies do not support that poudrage is any better than slurry [4-6]. Additionally, from a physiologic standpoint, it seems very likely that as soon as the operation is over, the chest tube is clamped, and the patient is awakened, any fluid remaining in the pleural space and any effusion that accumulates over the ensuing hours will rapidly redistribute the talc, as if it had been injected in a slurry. Another strategy is to frequently change the patient’s position while the chest tube is clamped, hoping that gravity will assist with dissemination of the talc. Although an attractive concept, studies indicate this does not improve results and that pleural circulation distributes an agent independent of positioning [14-16]. Better strategies are needed, and effectively addressing these problems should make pleurodesis a more attractive option.

Over the past decade, fibrinolytic therapy has proven an effective technique for eliminating pleural fluid loculations [10-12]. Fibrinolytics have also been given prior to pleurodesis to improve lung expansion and achieve a better result [17]. In a randomized controlled study of streptokinase compared to saline before TSP, Saydam and colleagues found that thrombolytic therapy was associated with improved lung expansion by CT imaging, decreased dyspneic symptoms and lower recurrence [18]. To the best of our knowledge, there is no report of using thrombolytics with and/or after the chemical pleurodesis agent.

The good experience we have had with tPA for management of complex pleural effusions led us to begin using tPA to promote drainage of loculated collections that developed after administration of talc slurry. Our early experience was promising and safe. Therefore, we considered the strategy of using tPA with TSP more deliberately and developed two hypotheses. First, best results with chemical pleurodesis are achieved when the sclerosing agent comes in contact with the greatest surface area of the mesothelial lining [19,20]. Fibrinolytics may enhance this process by breaking down small loculations to improve access of the agent to a greater surface area, and by lysing any fibrin coating that would inhibit direct contact of the agent with mesothelial cells. This hypothesis suggests that administration of tPA with talc slurry, not just before, would improve results. And second, because fibrinolytics effectively break down pleural fluid loculations, administration of tPA as these loculations develop during pleurodesis may contribute to a more uniform result. However, this effect may also inhibit pleurodesis. Pleural mesothelial cells normally produce urokinase and tPA, making the pleural space fibrinolytic. The inflammatory cascade induced by pleurodesis changes the pleural coagulation-fibrinolysis balance and causes the formation of fibrin links between the pleural surfaces. Fibroblasts proliferate in the fibrin mesh and produce collagen, creating a well-vascularized connective tissue bond between the visceral and parietal pleura [19-21]. This bond eliminates the pleural space, and thus prevents reaccumulation of pleural fluid. Administration of tPA during this process may eliminate developing loculations, but probably also degrades the fibrin mesh and prevents these last steps of pleurodesis from occurring. However, this effect is probably time-sensitive. The inflammation necessary for pleurodesis is likely an ongoing process over many days, so that a brief, properly timed period of fibrinolysis may eliminate developing loculations at a time when inflammation is continuing, allowing subsequent fibrin formation to complete pleurodesis.

To evaluate the first hypothesis, we looked specifically at cases in which tPA was given only with talc slurry. Results were promising, with some patients realizing a radiographic outcome that was better than before pleurodesis. Furthermore, with higher doses of tPA, no patient required repeat pleurodesis. Although maximum chest tube duration was very long, the median was less than when no tPA was used. The improvement in radiographic result and decreased need for repeat pleurodesis suggest that tPA may improve distribution of the pleurodesis agent and facilitate a more homogeneous pleurodesis.

To evaluate the second hypothesis, we looked at cases in which tPA was given after talc slurry. These results showed a high proportion of satisfactory radiographic results, with some patients better than before pleurodesis. The incidence of repeat pleurodesis, an indication that the tPA probably interfered with pleurodesis, was low when the tPA was given early after talc slurry (two to three days), but high when given at an interval of five days or more. These findings suggest that if fibrinolytic is given during an early window, loculated collections can be effectively drained while ongoing inflammation will continue the process of pleurodesis.

There are several other important findings in this review. First, tPA did not precipitate significant bleeding in any patient, either in the pleural space or as a result of systemic absorption, even in patients with malignant effusions. Our experience with these 60 cases is that this treatment is safe. Second, pigtail catheters rather than larger chest tubes were used in all patients, demonstrating that chest tube size is unimportant for effective drainage of the pleural space. And third, duration of chest tube drainage was exceptionally long in a few cases. This is a highly undesirable outcome and likely reflects the ad hoc approach towards chest tube management in these patients.

The important limitation to this study is that it is a retrospective review of a series of patients treated without a clear, prospective protocol. As a result, there is considerable variability in the dosing and timing of tPA from one patient to the next, in the criteria used to determine if repeat TSP was indicated, and when the chest tube could be removed.

## Conclusions

We believe definitive conclusions about efficacy are not reasonable and that statistical analyses are of questionable value given the variability in the dosing and timing of tPA from one patient to the next, in the criteria used to determine if repeat TSP was indicated, and when the chest tube could be removed. However, we also believe that these findings provide sufficient evidence to conclude that tPA with pleurodesis is safe and may improve results of TSP. As a result, we are undertaking a formal, prospective evaluation using an investigational protocol based on this experience. Unfortunately, talc is no longer available, but the underlying physiology should be the same regardless of agent. In the interval since talc became unavailable, we have been using doxycycline for pleurodesis and are undertaking a prospective evaluation with this agent.
